# Supplementary material for: Tying the knot: Unraveling the intricacies of the coronavirus frameshift pseudoknot
Source: PLoS Comput Biol. 2024 May 7;20(5):e1011787. doi: 10.1371/journal.pcbi.1011787 (PMC11108256; doi:10.1371/journal.pcbi.1011787)
Supplement: Attachment — Submitted filename: Trinity_Response_Reviewers_Tying_The_Knot.pdf [file pcbi.1011787.s002.pdf]

Response to Reviewers

April 19, 2024

We would like to thank the reviewers for constructive feedback and editors for their time and consideration of this work. We have revised the manuscript based on the request for revisions. Following the reviewer summary is our point-by-point response to the comments with additions to the manuscript in blue.

Reviewer 1

The work of Trinity, Stege, and Jabbari in PCOMPBIOL-D-23-02112 describes a method of using phylogenetic covariation to constrain what are ultimately thermodynamic predictions of RNA conformations that are crucial to the mechanics of programmed ribosomal frameshift -1 RNA dynamics in coronaviruses, especially SARS-CoV-2. This is a key problem in CoV biology and pathogenesis and not understood well enough to permit the use of pharmaceuticals or even meaningfully constrain the evolutionary recombination dynamics in a class of viruses that undergo rampant combination. These are important questions, and this manuscript makes useful and probably important predictions of RNA conformation, key to this biology. It builds on earlier publications using these methods by the authors and others. The inclusion of sequence covariation is a very nice aspect of the paper and is appropriately central to their discussion.

The main proof of their method is the identification of new RNA conformations that are very likely to be important to the function of -1 programmed riboomal frameshifting.

Before publication, the authors should make a number of changes to clarify various points and clean up the manuscript in several areas.

1. This is large and growing field, and the authors do a reasonable job of citing the literature, but there are probably several citations to add. Please see below.

2. Clarify some language about the presence of multiple conformations in RNA.

3. Clarify some language about energy versus free energy of the system. Be more explicit about the implied temperature of the 2kcal/mol cut-off, being both 1000K or 3.4% occupancy at 298K. This analysis is unlikely to be sensitive to conformations with less than $\sim 3\%$ occupancy at room temperature.

4. Are the alignments used to calculate covariation available? If not, please state why not.

5. Please provide a rough estimate of the run time for methods described here. A detailed comparison with molecular dynamics is not needed, because I expect a massive speed-up, in addition to the inclusion of important information missing from MD, but some quantification of run time would be helpful.

Most of the bulleted items below are probably minor to fix, a few might be a bit of work. The starred items are more important.

Response: We would like to thank the reviewer for their support of our work. In response to point 1, we have included additional citations as recommended. In response to point 2, we added information to clarify the details of multiple conformations. In response to point 3, we included the occupancy numbers as indicated. In response to the point 4, we added to coronavirus alignment subsection: "The alignment is used to calculate covariation via KnotAli, specifically, a set of pseudoknot-free base pairs is computed based on mutual information to guide subsequent free energy minimization." In response to point 5, we added runtime quantification. More details for all these responses to general points are specified in the line by line responses below.

- Aspects of this work looks like it builds (at least indirectly) on ideas from Sean Eddy and colleagues 10.1093/bioinformatics/btaa080 and Wilson and colleagues `https://pubmed.ncbi.nlm.nih.gov/10369773/`, so cites to those two papers may be in order.

  Response: Thank you for the suggestion. The citations were added to the revised manuscript (line 78).

- The last several sentences of the abstract should probably be shortened to the text in the Discussion: "Using a combination of thermodynamics methods and sequence covariation, our novel predictions suggest function of the attenuator hairpin via previously unknown pseudoknotted base pairing". Working in some version of, "While certain stems overlap, other pseudoknotted base pairs form which indicate potential conformational switching between the two structures." will nicely make the point that these sequence-informed analyses have an important message about the dynamics of -1 PRF.

  Response: We thank the reviewer for suggestions on moving important contributions to the abstract. The last two sentences are changed to the following: "Using a combination of thermodynamic methods and sequence covariation, our novel predictions suggest function of the attenuator hairpin via previously unknown pseudoknotted base pairing. While certain initial RNA folding is consistent, other pseudoknotted base pairs form which indicate potential conformational switching between the two structures."

- Line 4: Number of covid infections is clearly billions, not hundreds of millions. Please adjust intro text. `https://www.ncbi.nlm.nih.gov/pmc/articles/PMC9648705/`
  Response: While the number of real infections may be much higher than hundreds of millions and into the billions at this point, the confirmed numbers as indicated in the referenced article, is still in the hundreds of millions ($700,000,000+$). We revised the manuscript to " a vast multitude" in an attempt to avoid exact numbers.

- Line 9: 'frameshift event is elusive' for citation to the physical basis for -1PRF. Please cite some subset of: `https://www.ncbi.nlm.nih.gov/pmc/articles/PMC2670756/`, `https://pubmed.ncbi.nlm.nih.gov/18021801/`, and/or `https://pubmed.ncbi.nlm.nih.gov/25703095/`, and the authors really should consider also using their citation 21 `https://www.jbc.org/article/S0021-9258(17)50110-6/fulltext` to be also cited here.
  Response: Thank you for the suggestion. The citations were added to the revised manuscript (line 9).

- Line 21: "predicting secondary structures for the 68 nucleotide (nt) SARS-CoV-2 sequence" Give the coordinates in the genome or in ORF1ab for the 68 nt structure in the text to clarify.
  Response: The genome coordinates were added to the line 40 of the revised manuscript "(genome coordinates 13475 – 13542)"

- line 57: Most or all large-ish biomolecule conformational states are transient, and intermediate states is the correct terminology, please remove the "i.e." and "transient" from the sentence.
  Response: As per the reviewer's suggestion, "i.e." and "transient" were removed from the revised manuscript.

- *** Lines 60-62: "The RNA sequence itself is the primary factor in determining the structure of an RNA, ergo mutations to the frameshift element sequence can disrupt structure-function." This strong statement about RNA sequence should be moderated to give better context. Other physical effects aside from sequence play key roles in RNA structure. The fact that the authors are reporting a method that indirectly includes those non-sequence effects should be seen as progress for which they should be congratulated, but does not show other effects are nonexistent. `https://www.ncbi.nlm.nih.gov/pmc/articles/PMC10091408/` `https://pubs.acs.org/doi/epdf/10.1021/ja074191` etc. The rest of the paragraph is not invalidated or even weakened by correcting the first sentence in the paragraph.
  Response: We acknowledge that other factors such as ion concentration and interactions with other molecules can affect RNA structure. As such we revised the statement to "Mutations to the sequence of an RNA molecule can significantly disrupt structure-function".

- Line 74: Is the use of "cf." intended by the authors? `https://www.law.cornell.edu/wex/cf` Reading the text feels like they mean "i.e." or perhaps, "N.B."
  Response: Thank you for bringing this point to our attention. We replaced references to "c.f" in the revised manuscript with "see".

- *** Line 81: Please explain in more detail, "Each RNA loop (i.e., unpaired region closed by a base pair) is assigned an energy value." What energy? The pair-binding energy? Is that energy only a function of the pair, and not the sequence context? Or is it assigned a free energy (i.e. including an assigned entropy)? Are this energies in the supporting information, or in the author's earlier publications?
  Response: We revised the manuscript with more explanation on our energy model (see lines 82 to 89 of the revised manuscript).

- *** Line 86: "it is more expensive computationally" I assume this means more expensive than the method presented in this paper. Please be explicit about the comparison of "more expensive". Than what? The speed of these calculations feels like it should be a significant advantage of this method over all-atom simulation, especially compared with atomistic solvent. Some rough quantification of the computation time of this method would be helpful.
  Response: We revised the manuscript as follows "however, it is more expensive computationally than our Shapify method ($\sim$ 3 seconds for structure prediction at sequence length 222 nt)."

- Line 88: "cf." is defined here: `https://www.law.cornell.edu/wex/cf|`. I assume the authors mean "note well", or in Latin, "Nota Bene," "N.B."
  Response: Thank you for bringing this point to our attention. We replaced references to "N.B." in the revised manuscript with "see".

- Line 121: Please cite `https://pubmed.ncbi.nlm.nih.gov/20159159/` `https://www.science.org/doi/abs/10.1126/science.1749933` for the text, "landscape of energetically favourable RNA structures," to clarify the standard but confusing terminology of "native pseudoknot". While it is clear that the authors understand the point about multiple conformations made by the two citations above, readers who are less familiar with physical chemistry and biological physics will benefit from the citations showing that "native state basin" is correct terminology while "native state" is wrong.
  Response: Thank you for the suggestion. We added the suggested citations ([65, 66]) to our revised manuscript.

- Line 161 "find the MFE (most stable) structure for a 161 given sequence S" There is no requirement that the ground state of a system be unique (meaning in the limit of zero temperature). Degenerate ground states (where the objective function being minimize is the energy, not the free energy) are possible depending on system symmetry, including polymers and other glassy systems. A non-zero temperature, there can be many effectively degenerate ground states that – in a coarse-grained/non-microcanonical model of polymer conformation as being used in this paper – are best characterized by the system's entropy in a free energy of the system. I realize that the terminiology is "standard", but it is problematic. Please do what you can in your discussion to clarify.
  Response: We clarified the text both in the Introduction (lines 82-88) and Materials and Methods, Section 2.1 (lines 157-159).

- line 168 "SARS-CoV-2 secondary structure prediction efforts find multiple length-dependent 168 structural motifs related to the frameshift event" I believe it would be clearer to write something like, "SARS-CoV-2 secondary structure prediction efforts find structural motifs of several lengths that related to the frameshift event"
  Response: Thank you for the suggestion. The text was revised accordingly.

- line 173: The use of "cf." is extraneous here. Translated from Latin, "cf." means "compare". Most likely the authors mean "q.v.". For further specifics, please see, `https://writingcenter.unc.edu/tips-and-tools/latin-terms-and-abbreviations/`
  Response: We replaced references to "c.f" in the revised manuscript with "see". Thank you!

- Figure 2 caption: Do the authors mean the free energy, or the energy. Unless there is an implicit temperature and ion concentration in their calculations, then these are either (i) energies, not free energies, or (ii) the free energy is there, but possibly not completely well-defined as a computational expediency that allows for implicit inclusion as sequence information via a sequence alignment to known or predicted RNA structures. Later in the text, it is clearly free energy.
  Response: We corrected references to "energy" in the manuscript to "free energy" and added clarification on the temperature and ionic concentration used in our energy model.

- Line 177: Is it possible that this text is making it unclear what is found in this analysis, in particular: windows lengths of RNA, versus what is actually present in the ensemble of RNA structures? If so, please clarify the wording.
  Response: We revised the text as follows "Dual graph representations allow for variable length of stems and loops, which makes RNA secondary structure pattern or motif identification within the ensemble of RNA structures easier."

- Line 214: Seems like there should be a way to work in a reference to `https://cshperspectives.cshlp.org/content/10/10/a032433.full` for "KnotAli [53] combines the strengths of MFE prediction and alignment-based methods through relaxed hierarchical folding. KnotAli uses a multiple RNA sequence alignment as input to predict possibly pseudoknotted secondary structures for each sequence in the alignment." This history of RNA folding as been a long one, and citing a more colloquial discussion of that history will probably help the interested non-specialist reader.
  Response: We revised the text to "KnotAli [64] combines the strengths of MFE prediction and alignment-based methods through relaxed hierarchical folding (see [71] for colloquial discussion of RNA folding history)"; lines 225-226 of the revised manuscript.

- *** Line 228: "With respect to the four structures from the four methods, we include in our results any additional suboptimal structures that fall within 2 kcal/mol of the MFE prediction." This is the first mention of a temperature scale I see in the paper. The free energy calculation are thus at 1000K, meaning that at 298K (room T), the calculation accepts minority populations down to 3.4% occupancy. Please spell this out. Here is an on-line converter: `https://www.colby.edu/chemistry/PChem/Hartree.html`
  Response: Thank you for the suggestion. We revised the text as follows: "In addition to the MFE structure we include any additional suboptimal structures resulting from the four methods that fall within 2 kcal/mol of the MFE structure in our results. Given the energy model used in our work (that of HotKnots V2.0 [56]) is based on temperature of 37$\circ$C (310.15 K), a 2 kcal/mol temperature scale accepts minority populations down to 3.75% occupancy or roughly a difference of two or three base pairs." See lines 241-246 of the revised manuscript.

- Line 230: "and identified up to 20 lowest free energy unique stems" This implies a cut-off of 5% occupancy for roughly equi-probable conformations. For other distributions of occupancy/probability, it is unclear to me exactly what occupancy of the 20th state would be. But in any event, this is a heuristic that isn't too far off from the 3% occupancy above.
  Response: To clarify the point we added the following to the revised manuscript: "Assuming equi-probable RNA conformations, this cut-off is similar to the 3.75% occupancy threshold above." See lines 250-251 of the revised manuscript.

- * Line 234: Section 2.5 Data. "We obtained the coronavirus alignment of Schlick et al. [33], where out of 3760 SARS-CoV-2 coronavirus sequences [38], and 2855 other coronavirus sequences [62], 1248 sequences were found to be non-redundant [63]. These 1248 sequences were structurally aligned to the 222 nt SARS-CoV-2 frameshift element SHAPE consensus structure [33] using the Infernal covariance model [64] giving a final result of 182 non-duplicate homologous sites including seven SARS-CoV-2 sequences." This describes a major part of the methodology for why this approach is successful, and naming the subsection "Data" is an odd choice. The way the authors do their alignment here is a big part of the story: aligning to an RNA-world functional element. Please clarify the section headers. Is the alignment available as supporting information? Also R1: It isn't clear where the sequence alignment resides. Perhaps GISAID rules prohibit direct publication.

Response: As per the reviewer suggestion we revised the subsection heading from 'Data' to 'Coronaviruses alignment and sequence information'. We further added alignment and source information to the GitHub: `https://github.com/ltrinity/TyingTheKnot`.

- * Figure 4's red diamonds are essentially illegible. The figure should be re-made so that it can be studied and conveys the key information. No tiny symbols, please. The "pink" color looks purple to me.
  Response: We revised Figure 4 and updated the caption to "Mutations are indicated with red ovals. A $\triangle$ symbol represents the mutation from the nucleotide on the left to the nucleotide on the right." References to 'pink' were changed to 'fuchsia' throughout.

- * Figure 5. Same comments as for Fig. 4.
  Response: We revised Figure 5 and updated the caption to "Mutations are indicated with red ovals. A $\triangle$ symbol represents the mutation from the nucleotide on the left to the nucleotide on the right."

- * Page 10/21: Please clearly state what was done with the conformations in Fig. 6 that are grey. Where they included or not included in further analysis leading to, for example figure 3 and 4 and 8-10?
  Response: We revised caption of Fig. 6 for clarification to "Each dot represents a Shapify predicted secondary structure for the SARS-CoV-2 frameshift sequence (independent from KnotAli predictions, see Section 2.6, Table 1). X-axis represents window size, y-axis represents free energy per nt. Dots are colored based on the four listed dual-graph motifs (legend in top-right) detected at or directly 3$'$ of the slippery sequence (see Table 2), or grey for others. Noteworthy structures not matched with a motif (grey dots here) are visualized in Fig 8-10."

- * Figures 8, 9 , and 10: the different window lengths give rather different RNA conformations. The discussion of this section, "While certain stems overlap, other pseudoknotted base pairs form which indicate potential conformational switching between the two structures." is also one of the authors highlights.
  Response: Thank you for highlighting this point. We have added this contribution to the end of the abstract in the revised manuscript.

- *** Line 405: "Our novel predictions suggest function of the attenuator hairpin via previously unknown pseudoknotted base pairing, paving the way for future tertiary modeling of SARS-CoV-2 frameshift RNA structure-function." This is major message of the paper, but by my reading, this result is buried. Please repeat this text in a prominent location in the text.
  Response: Thank you for the suggestion. As stated above we have added this contribution to the end of the abstract in the revised manuscript.

- Line 419: "Understanding how initial most stable stems give way" Should be, "Understanding how initial (most stable stems) give way"
  Response: We revised the text accordingly.

References

[1] Chen Y, Liu Q, Guo D. Emerging coronaviruses: genome structure, replication, and pathogenesis. J Med Virol. 2020;92(4):418–423.

[2] Atkins JF, Loughran G, Bhatt PR, Firth AE, Baranov PV. Ribosomal frameshifting and transcriptional slippage: From genetic steganography and cryptography to adventitious use. Nucleic Acids Res. 2016;44(15):7007–7078.

[3] Dinman JD. Mechanisms and implications of programmed translational frameshifting. Wiley Interdiscip Rev: RNA. 2012;3(5):661–673.

[4] Riegger RJ, Caliskan N. Thinking outside the frame: impacting genomes capacity by programmed ribosomal frameshifting. Front Mol Biosci. 2022;9:842261.

[5] Roman C, Lewicka A, Koirala D, Li NS, Piccirilli JA. The SARS-CoV-2 programmed- 1 ribosomal frameshifting element crystal structure solved to 2.09 Å using chaperone-assisted RNA crystallography. ACS Chem Biol. 2021;16(8):1469–1481.

[6] Giedroc DP, Cornish PV. Frameshifting RNA pseudoknots: structure and mechanism. Virus research. 2009;139(2):193–208.

[7] Green L, Kim CH, Bustamante C, Tinoco Jr I. Characterization of the mechanical unfolding of RNA pseudoknots. J Mol Biol. 2008;375(2):511–528.

[8] Yan S, Wen JD, Bustamante C, Tinoco I. Ribosome excursions during mRNA translocation mediate broad branching of frameshift pathways. Cell. 2015;160(5):870–881.

[9] Kelly JA, Olson AN, Neupane K, Munshi S, San Emeterio J, Pollack L, et al. Structural and functional conservation of the programmed- 1 ribosomal frameshift signal of SARS coronavirus 2 (SARS-CoV-2). J Biol Chem. 2020;295(31):10741–10748.

[10] Bhatt PR, Scaiola A, Loughran G, Leibundgut M, Kratzel A, Meurs R, et al. Structural basis of ribosomal frameshifting during translation of the SARS-CoV-2 RNA genome. Science. 2021;372(6548):1306–1313.

[11] Lan TC, Allan MF, Malsick LE, Woo JZ, Zhu C, Zhang F, et al. Secondary structural ensembles of the SARS-CoV-2 RNA genome in infected cells. Nat Commun. 2022;13(1):1128.

[12] Zhang K, Zheludev IN, Hagey RJ, Haslecker R, Hou YJ, Kretsch R, et al. Cryo-EM and antisense targeting of the 28-kDa frameshift stimulation element from the SARS-CoV-2 RNA genome. Nat Struct Mol Biol. 2021;28(9):747–754.

[13] Yan S, Zhu Q, Hohl J, Dong A, Schlick T. Evolution of coronavirus frameshifting elements: Competing stem networks explain conservation and variability. PNAS. 2023;120(20):e2221324120.

[14] Kelly JA, Woodside MT, Dinman JD. Programmed- 1 ribosomal frameshifting in coronaviruses: a therapeutic target. Virology. 2021;554:75–82.

[15] Yang M, Olatunji FP, Rhodes C, Balaratnam S, Dunne-Dombrink K, Seshadri S, et al. Discovery of Small Molecules Targeting the Frameshifting Element RNA in SARS-CoV-2 Viral Genome. ACS Med Chem Lett. 2023;.

[16] Varricchio C, Mathez G, Pillonel T, Bertelli C, Kaiser L, Tapparel C, et al. Geneticin shows selective antiviral activity against SARS-CoV-2 by interfering with programmed-1 ribosomal frameshifting. Antiviral Research. 2022;208:105452.

[17] Mathez G, Cagno V. Small Molecules Targeting Viral RNA. Int J Mol Sci. 2023;24(17):13500.

[18] Neupane K, Munshi S, Zhao M, Ritchie DB, Ileperuma SM, Woodside MT. Anti-frameshifting ligand active against SARS coronavirus-2 is resistant to natural mutations of the frameshift-stimulatory pseudoknot. J Mol Biol. 2020;432(21):5843–5847.

[19] Sun Y, Abriola L, Niederer RO, Pedersen SF, Alfajaro MM, Monteiro VS, et al. Restriction of SARS-CoV-2 replication by targeting programmed- 1 ribosomal frameshifting. PNAS. 2021;118(26).

[20] Kurreck J, Wyszko E, Gillen C, Erdmann VA. Design of antisense oligonucleotides stabilized by locked nucleic acids. Nucleic Acids Res. 2002;30(9):1911–1918.

[21] Munshi S, Neupane K, Ileperuma SM, Halma MT, Kelly JA, Halpern CF, et al. Identifying inhibitors of-1 programmed ribosomal frameshifting in a broad spectrum of coronaviruses. Viruses. 2022;14(2):177.

[22] Ahn DG, Yoon GY, Lee S, Ku KB, Kim C, Kim KD, et al. A Novel Frameshifting Inhibitor Having Antiviral Activity against Zoonotic Coronaviruses. Viruses. 2021;13(8):1639.

[23] Yu D, Han HJ, Yu J, Kim J, Lee GH, Yang JH, et al. Pseudoknot-targeting Cas13b combats SARS-CoV-2 infection by suppressing viral replication. Mol Ther. 2023;31(6):1675–1687.

[24] Petrosillo N, Viceconte G, Ergonul O, Ippolito G, Petersen E. COVID-19, SARS and MERS: are they closely related? Clin Microbiol Infect. 2020;.

[25] Trinity L, Wark I, Lansing L, Jabbari H, Stege U. Shapify: Paths to SARS-CoV-2 frameshifting pseudoknot. PLoS Comput Biol. 2023;19(2):e1010922.

[26] Sekar RV, Oliva PJ, Woodside MT. Modelling the structures of frameshift-stimulatory pseudoknots from representative bat coronaviruses. PLoS Comput Biol. 2023;19(5):e1011124.

[27] Ritchie DB, Foster DA, Woodside MT. Programmed- 1 frameshifting efficiency correlates with RNA pseudoknot conformational plasticity, not resistance to mechanical unfolding. PNAS. 2012;109(40):16167–16172.

[28] Ritchie DB, Soong J, Sikkema WK, Woodside MT. Anti-frameshifting ligand reduces the conformational plasticity of the SARS virus pseudoknot. J Am Chem Soc. 2014;136(6):2196–2199.

[29] Wu B, Zhang H, Sun R, Peng S, Cooperman BS, Goldman YE, et al. Translocation kinetics and structural dynamics of ribosomes are modulated by the conformational plasticity of downstream pseudoknots. Nucleic Acids Res. 2018;46(18):9736–9748.

[30] Neupane K, Zhao M, Lyons A, Munshi S, Ileperuma SM, Ritchie DB, et al. Structural dynamics of single SARS-CoV-2 pseudoknot molecules reveal topologically distinct conformers. Nat Commun. 2021;12(1):1–9.

[31] Pekarek L, Zimmer MM, Gribling-Burrer AS, Buck S, Smyth R, Caliskan N. Cis-mediated interactions of the SARS-CoV-2 frameshift RNA alter its conformations and affect function. Nucleic Acids Res. 2023;51(2):728–743.

[32] Siegfried NA, Busan S, Rice GM, Nelson JA, Weeks KM. RNA motif discovery by SHAPE and mutational profiling (SHAPE-MaP). Nat Med. 2014;11(9):959–965.

[33] Hajdin CE, Bellaousov S, Huggins W, Leonard CW, Mathews DH, Weeks KM. Accurate SHAPE-directed RNA secondary structure modeling, including pseudoknots. PNAS. 2013;110(14):5498–5503.

[34] Huston NC, Wan H, Strine MS, Tavares RdCA, Wilen CB, Pyle AM. Comprehensive in vivo secondary structure of the SARS-CoV-2 genome reveals novel regulatory motifs and mechanisms. Mol Cell. 2021;81(3):584–598.

[35] Manfredonia I, Nithin C, Ponce-Salvatierra A, Ghosh P, Wirecki TK, Marinus T, et al. Genome-wide mapping of SARS-CoV-2 RNA structures identifies therapeutically-relevant elements. Nucleic Acids Res. 2020;48(22):12436–12452.

[36] Yang SL, DeFalco L, Anderson DE, Zhang Y, Aw JGA, Lim SY, et al. Comprehensive mapping of SARS-CoV-2 interactions in vivo reveals functional virus-host interactions. Nat Commun. 2021;12(1):1–15.

[37] Schlick T, Zhu Q, Dey A, Jain S, Yan S, Laederach A. To knot or not to knot: multiple conformations of the SARS-CoV-2 frameshifting RNA element. J Am Chem Soc. 2021;143(30):11404–11422.

[38] Omar SI, Zhao M, Sekar RV, Moghadam SA, Tuszynski JA, Woodside MT. Modeling the structure of the frameshift-stimulatory pseudoknot in SARS-CoV-2 reveals multiple possible conformers. PLoS Comput Biol. 2021;17(1):e1008603.

[39] He W, San Emeterio J, Woodside MT, Kirmizialtin S, Pollack L. Atomistic structure of the SARS-CoV-2 pseudoknot in solution from SAXS-driven molecular dynamics. Nucleic Acids Res. 2023; p. gkad809.

[40] Wang X, Tan YL, Yu S, Shi YZ, Tan ZJ. Predicting 3D structures and stabilities for complex RNA pseudoknots in ion solutions. Biophys J. 2023;122(8):1503–1516.

[41] Schlick T, Zhu Q, Jain S, Yan S. Structure-altering mutations of the SARS-CoV-2 frameshifting RNA element. Biophys J. 2021;120(6):1040–1053.

[42] Shu Y, McCauley J. GISAID: Global initiative on sharing all influenza data–from vision to reality. Eurosurveillance. 2017;22(13):30494.

[43] Chen AT, Altschuler K, Zhan SH, Chan YA, Deverman BE. COVID-19 CG enables SARS-CoV-2 mutation and lineage tracking by locations and dates of interest. Elife. 2021;10:e63409.

[44] Fourmy D, Yoshizawa S. A cytosine-to-uracil change within the programmed-1 ribosomal frameshift signal of SARS-CoV-2 results in structural similarities with the MERS-CoV signal. Cold Spring Harbor Laboratory; 2020.

[45] Tinoco Jr I, Bustamante C. How RNA folds. J Mol Biol. 1999;293(2):271–281.

[46] Wilkinson KA, Merino EJ, Weeks KM. RNA SHAPE chemistry reveals nonhierarchical interactions dominate equilibrium structural transitions in tRNAAsp transcripts. J Am Chem Soc. 2005;127(13):4659–4667.

[47] Ding F, Sharma S, Chalasani P, Demidov VV, Broude NE, Dokholyan NV. Ab initio RNA folding by discrete molecular dynamics: from structure prediction to folding mechanisms. RNA. 2008;14(6):1164–1173.

[48] Cho SS, Pincus DL, Thirumalai D. Assembly mechanisms of RNA pseudoknots are determined by the stabilities of constituent secondary structures. PNAS. 2009;106(41):17349–17354.

[49] Chen G, Chang KY, Chou MY, Bustamante C, Tinoco I. Triplex structures in an RNA pseudoknot enhance mechanical stability and increase efficiency of–1 ribosomal frameshifting. PNAS. 2009;106(31):12706–12711.

[50] Schroeder SJ, Turner DH. Optical melting measurements of nucleic acid thermodynamics. In: Methods Enzymol.. vol. 468. Elsevier; 2009. p. 371–387.

[51] Turner DH, Sugimoto N, Freier SM. RNA structure prediction. Annu Rev Biophys and Biochem. 1988;17(1):167–192.

[52] Mathews DH, Sabina J, Zuker M, Turner DH. Expanded sequence dependence of thermodynamic parameters improves prediction of RNA secondary structure. J Mol Biol. 1999;288(5):911–940.

[53] Mathews DH, Disney MD, Childs JL, Schroeder SJ, Zuker M, Turner DH. Incorporating chemical modification constraints into a dynamic programming algorithm for prediction of RNA secondary structure. PNAS. 2004;101(19):7287–7292.

[54] Andronescu MS, Pop C, Condon AE. Improved free energy parameters for RNA pseudoknotted secondary structure prediction. RNA. 2010;16(1):26–42.

[55] Wang J, Wang J, Huang Y, Xiao Y. 3dRNA v2. 0: An Updated Web Server for RNA 3D Structure Prediction. Int J Mol Sci. 2019;20(17):4116.

[56] Zok T, Antczak M, Zurkowski M, Popenda M, Blazewicz J, Adamiak RW, et al. RNApdbee 2.0: multifunctional tool for RNA structure annotation. Nucleic Acids Res. 2018;46(W1):W30–W35.

[57] Li S, Olson WK, Lu XJ. Web 3DNA 2.0 for the analysis, visualization, and modeling of 3D nucleic acid structures. Nucleic Acids Res. 2019;47(W1):W26–W34.

[58] Biesiada M, Purzycka KJ, Szachniuk M, Blazewicz J, Adamiak RW. Automated RNA 3D structure prediction with RNAComposer. In: RNA Structure Determination. Springer; 2016. p. 199–215.

[59] Parisien M, Major F. The MC-Fold and MC-Sym pipeline infers RNA structure from sequence data. Nature. 2008;452(7183):51–55.

[60] Krokhotin A, Houlihan K, Dokholyan NV. iFoldRNA v2: folding RNA with constraints. Bioinformatics. 2015;31(17):2891–2893.

[61] Li B, Cao Y, Westhof E, Miao Z. Advances in RNA 3D structure modeling using experimental data. Front Genet. 2020;11:574485.

[62] Gray M, Chester S, Jabbari H. KnotAli: informed energy minimization through the use of evolutionary information. BMC Bioinf. 2022;23(1):159.

[63] Deng NJ, Cieplak P. Free energy profile of RNA hairpins: a molecular dynamics simulation study. Biophys J. 2010;98(4):627–636.

[64] Frauenfelder H, Sligar SG, Wolynes PG. The energy landscapes and motions of proteins. Science. 1991;254(5038):1598–1603.

[65] Yan S, Zhu Q, Jain S, Schlick T. Length-dependent motions of SARS-CoV-2 frameshifting RNA pseudoknot and alternative conformations suggest avenues for frameshifting suppression. Nat Commun. 2022;13(1):4284.

[66] Fera D, Kim N, Shiffeldrim N, Zorn J, Laserson U, Gan HH, et al. RAG: RNA-As-Graphs web resource. BMC Bioinf. 2004;5(1):1–9.

[67] Gan HH, Pasquali S, Schlick T. Exploring the repertoire of RNA secondary motifs using graph theory; implications for RNA design. Nucleic Acids Res. 2003;31(11):2926–2943.

[68] Zhu Q, Petingi L, Schlick T. RNA-As-Graphs Motif Atlas—Dual Graph Library of RNA Modules and Viral Frameshifting-Element Applications. Int J Mol Sci. 2022;23(16):9249.

[69] Herschlag D, Bonilla S, Bisaria N. The story of RNA folding, as told in epochs. Cold Spring Harbor Perspect Biol. 2018;10(10):a032433.

[70] Pickett BE, Sadat EL, Zhang Y, Noronha JM, Squires RB, Hunt V, et al. ViPR: an open bioinformatics database and analysis resource for virology research. Nucleic Acids Res. 2012;40(D1):D593–D598.

[71] Fu L, Niu B, Zhu Z, Wu S, Li W. CD-HIT: accelerated for clustering the next-generation sequencing data. Bioinformatics. 2012;28(23):3150–3152.

[72] Nawrocki EP, Eddy SR. Infernal 1.1: 100-fold faster RNA homology searches. Bioinformatics. 2013;29(22):2933–2935.

[73] Brister JR, Ako-Adjei D, Bao Y, Blinkova O. NCBI viral genomes resource. Nucleic Acids Res. 2015;43(D1):D571–D577.

[74] Trinity L, Will S, Ponty Y, Stege U, Jabbari H. CParty: Conditional partition function for density-2 RNA pseudoknots. bioRxiv. 2023; p. 2023–05.

[75] Plant EP, Pérez-Alvarado GC, Jacobs JL, Mukhopadhyay B, Hennig M, Dinman JD. A three-stemmed mRNA pseudoknot in the SARS coronavirus frameshift signal. PLoS Biol. 2005;3(6):e172.

[76] Rehfeld F, Eitson JL, Ohlson MB, Chang TC, Schoggins JW, Mendell JT. CRISPR screening reveals a dependency on ribosome recycling for efficient SARS-CoV-2 programmed ribosomal frameshifting and viral replication. Cell Rep. 2023;42(2).

[77] Sato K, Kato Y, Hamada M, Akutsu T, Asai K. IPknot: fast and accurate prediction of RNA secondary structures with pseudoknots using integer programming. Bioinformatics. 2011;27(13):i85–i93.

[78] Ren J, Rastegari B, Condon A, Hoos HH. HotKnots: heuristic prediction of RNA secondary structures including pseudoknots. RNA. 2005;11(10):1494–1504.

[79] Knizhnik E, Chumakov S, Svetlova J, Pavlova I, Khodarovich Y, Brylev V, et al. Unwinding the SARS-CoV-2 Ribosomal Frameshifting Pseudoknot with LNA and G-Clamp-Modified Phosphorothioate Oligonucleotides Inhibits Viral Replication. Biomol. 2023;13(11):1660.
